# Acceleration of mass transfer processes in plants due to the geometric structure: a fractional order equation of mass transfer and its application

**DOI:** 10.1038/s41598-019-51362-y

**Published:** 2019-10-30

**Authors:** V. E. Arkhincheev

**Affiliations:** 1 Laboratory of Applied Physics, Advanced Institute of Materials Science, Ton Duc Thang University, Ho Chi Minh City, Vietnam; 2 Faculty of Applied Sciences, Ton Duc Thang University, Ho Chi Minh City, Vietnam

**Keywords:** Plant evolution, Statistical physics, thermodynamics and nonlinear dynamics

## Abstract

The problem of mass transfer in living plants in the framework of the comb model was studied. The fractional order equation for problem of mass transfer was deduced and its application for transfer in the plants was considered. The different temporal asymptotic, which occurred due to geometry of plants, were obtained. It was established that mass transfer processes in living plants depend on the geometric structure of plants, namely, it is mass transfer is accelerated from steam to branches. The discussion of obtained results was given.

## Introduction

Recently mass transfer processes in porous media with a complex structure are intensively studied. Numerous studies have shown that diffusion in such a media has an anomalous character that is the anomalous dependence of the root-mean-square displacement of diffusing particles on time appears^1–3^ and it cannot be described by the classical diffusion equation. It also defines a new self-similar behavior, as well as a non-Gaussian form for a stable distribution of diffusing particles^4^. For the quantitative description of anomalous diffusion of particles in porous materials the method of fractional calculus was used^5–7^. However, till recently, the diffusion transport was studied on exact fractal structures as Serpinskii gaskets or on solid-state structures having a fractal porous structure^8,9^.

In this work, we study the mass transfer processes in living plants, which, in our opinion, have an internal fractal structure of pores through which water and nutrients are transferred to ensure the growth of these plants. The fractional calculus approach was applied for mass transfer problem in plants.

As a three-dimensional model of plants, a mathematical model of a comb-like structure was used, consisting of a well-conducting axis - the stem of a plant and two-dimensional ribs attached to it, analogous to branches. Earlier, this model was introduced in^10–13^ and it was used to describe electron transport in strongly disordered media and other problems as convection in disordered sytems too^14^.

The article is structured as follows. In Section 2 the comb model was introduced. In Section 3 the effective fractional order three-dimensional equation of mass transfer was obtained. In Section 4 the brief description of mathematical method –fractional calculus, which used in the paper, was given. The Section 5 concludes the paper and the features of mass transfer in plants objects are discussed.

## Three-Dimensional Model of Comb Structure as a Plant Model

**Figure 1:**
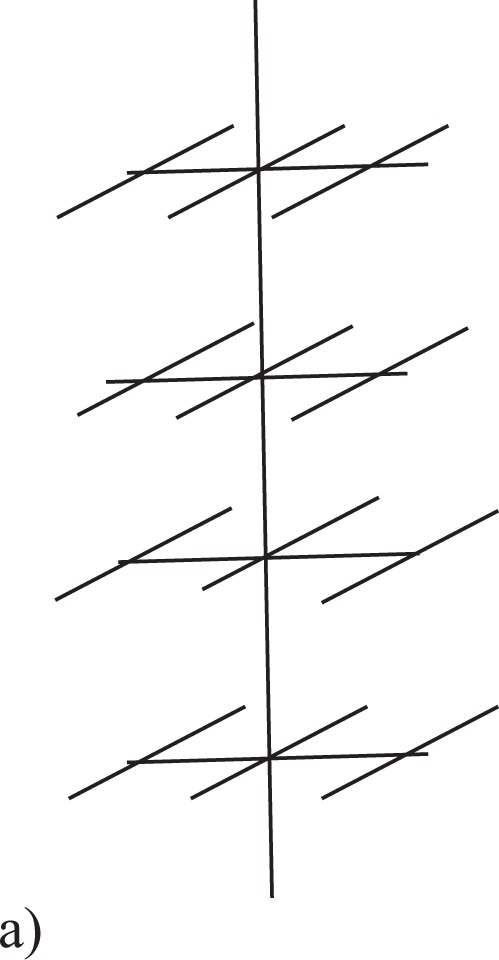
The comb model as a mathematical model of plant.

The standard model of the two-dimensional comb structure consists of a well conducting axis — a conducting channel (similar to the skeleton of a percolation cluster) and fingers attached to the axis. The three-dimensional comb structure is formed by attaching additional perpendicular fingers in the Z direction to the fingers - see Fig. 1.

The feature of diffusion on the comb structure is the possibility of displacement along *X* direction only along the intersection of the planes *Y* = 0 and *Z* = 0. In other words, the diffusion coefficient is different from zero only when *Y* = 0 and *Z* = 0:

1 $${D}_{xx}={D}_{1}\delta (y)\delta (z)$$

Similarly, we obtain that motion along the *Y*- direction is possible only along the planes *Z* = 0. In other words, the diffusion coefficient is different from zero only when *Z* = 0:

2 $${D}_{yy}={D}_{2}\delta (z).$$

Diffusion along the *Z*-direction of fingers of the structure is of the usual nature: *D*_*zz*_ = *D*_3_. Thus, we obtain the diffusion tensor:

3 $${D}_{ij}=(\begin{array}{ccc}{D}_{1}\delta (y)\delta (z) & 0 & 0\\ 0 & {D}_{2}\delta (z) & 0\\ 0 & 0 & {D}_{3}\end{array}).$$

Using the Fick law with the diffusion tensor (3) ${\overrightarrow{J}}_{d}=-\,\hat{D}\,\overrightarrow{\nabla }N$ and the equation of continuity, we obtain the diffusion equation:

4 $$[\frac{\partial }{\partial t}-{D}_{1}\delta (y)\delta (z)\frac{{\partial }^{2}}{\partial {x}^{2}}-{D}_{2}\delta (z)\frac{{\partial }^{2}}{\partial {y}^{2}}-{D}_{3}\frac{{\partial }^{2}}{\partial {z}^{2}}]G(x,y,z,t)=\delta (t)\delta (x)\delta (y)\delta (z)$$

Here *G*(*x*, *y*, *z*, *t*) is the Green’s function of the diffusion equation. A point source *δ*(*x*)*δ*(*y*)*δ*(*z*)*δ*(*t*) is used as the initial data. For further convenience, we made the Laplace transform with respect to time *t* and the Fourier transform with respect to the *X* coordinate:

5 $$[s+{D}_{1}{k}^{2}\delta (y)\delta (z)-{D}_{2}\delta (z)\frac{{\partial }^{2}}{\partial {y}^{2}}-{D}_{3}\frac{{\partial }^{2}}{\partial {z}^{2}}]G(k,y,z,s)=\delta (y)\delta (z)$$

The solution of Eq. (5) will be search in the form:

6 $$G(k,y,z,s)=g(k,s)\exp (-{\lambda }_{y}|y|-{\lambda }_{z}|z|).$$

Substituting (6) into (5), we define the parameters *λ*_*y*_, *λ*_*z*_ and the function *g*(*k*, *s*):

7 $$\begin{array}{ccc}{\lambda }_{z}=\sqrt{\frac{s}{{D}_{3}}},{\lambda }_{y}=\sqrt{\frac{2{D}_{3}{\lambda }_{z}}{{D}_{2}}} & , & g(k,s)=\frac{1}{2{D}_{2}{\lambda }_{y}+{D}_{1}{k}^{2}}\end{array}.$$

It is easy to verify that the root-mean-square displacement along the structure X is:

8 $$\langle {x}^{2}(t)\rangle \propto {(t)}^{1/4}.$$

Diffusion along the edges of the comb structure along the *Y*- direction is also anomalous:

9 $$\langle {y}^{2}(t)\rangle \propto {(t)}^{1/2}.$$

The displacement along the edges in the Z-direction is of ordinary nature:

10 $$\langle {z}^{2}(t)\rangle \propto t.$$

The obtained results lead to an interesting conclusion about the peculiarities of mass transfer in plants. Let us consider in more detail on the example of the migration of fertilizers introduced into the soil at the base of the plants. According to the results obtained above, the migration of fertilizers along the stem of the plant (in our notation along the structure axis — along the *X* direction) is of a slow nature — formula (8). This is easy to understand, because before they move along the trunk (axis of the comb-shaped structure), the particles diffuse into the lateral branches of the first generation (*Y*-direction), which slows down the diffusion front. It should be noted that the displacement along the branches of the first generation (in the *Y* direction) is also of a delayed character, described by formula (9). And only then begins the migration through the usual diffusion along the lateral branches of the second generation (in the *Z* - direction) – formula (10).

## N-Dimensional Stretched Comb Structure as a Model of Branched Plants

It is interesting to note that in the N-dimensional case, the comb model can be used as a model of a branched plant (tree) with branches of the (N − 1) generation (the axis corresponds to the stem of the plant, the fingers correspond to the branches of the (N − 1) generation). Let’s study to the N-dimensional case of the comb model. In this case, the diffusion tensor is described by the matrix:

11 $${D}_{ij}=(\begin{array}{ccccc}{D}_{1}\delta ({x}_{1})\mathrm{..}\delta ({x}_{n}) & 0 & \mathrm{...} & 0 & 0\\ 0 & {D}_{2}\delta ({x}_{2})\mathrm{..}\delta ({x}_{n}) & 0 & 0 & 0\\ \ldots  & \ldots  & \ldots  & \ldots  & \ldots \\ 0 & 0 & 0 & {D}_{n-1}\delta ({x}_{n-^{\prime} 1}) & 0\\ 0 & 0 & 0 & 0 & {D}_{n}\end{array}).$$

Accordingly, the solution of the N-dimensional diffusion problem will be sought in the form:

12 $$G(k,{x}_{2},{x}_{3}\ldots {x}_{n},s)=g(k,s)\exp (-{\lambda }_{2}|{x}_{2}|-{\lambda }_{3}|{x}_{3}|-\ldots -{\lambda }_{n}|{x}_{n}|).$$

The parameters are determined by the relations:

13 $$\begin{array}{ccc}{\lambda }_{n}^{2}=\frac{s}{{D}_{n}}, & {\lambda }_{n-1}^{2}=\frac{2{\lambda }_{n}{D}_{n}}{{D}_{n-1}}\ldots  & {\lambda }_{2}^{2}=\frac{2{\lambda }_{3}{D}_{3}}{{D}_{2}}\end{array}.$$

The function *g*(*s*, *k*) is defined in (7). Expressions (12) and (13) give a complete solution to the problem. For example, it is easy to calculate the root-mean-square displacement along the main axis structures:

14 $$\langle {X}_{1}^{2}(t)\rangle \propto {(t)}^{1/2(N-1)}.$$

The root-mean-square displacement for the next side fingers, which together with the attached fingers forms a (N − l) -dimensional comb structure, is equal to:

15 $$\langle {X}_{2}^{2}(t)\rangle \propto {(t)}^{1/2(N-2)}.$$

and so on. On the penultimate axis, from which only fingers of infinite length depart, we obtain:

16 $$\langle {X}_{N-1}^{2}(t)\rangle \propto {(t)}^{1/2}.$$

And at last fingers the usual diffusion has observed:

17 $$\langle {X}_{N}^{2}(t)\rangle \propto t.$$

**Figure 2:**
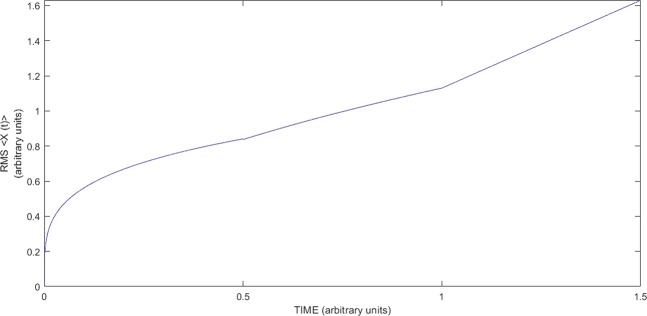
The change of temporal dependence of RMS displacement of mass transfer from stream to the branches of 1 and 2 generations.

Thus, a random walk on a multidimensional comb structure has a hierarchical nature, and many variants of the behavior of the root-mean-square displacement along the structure axes are arise – see Fig. 2.

## Method – Fractional order Diffusion Equations

Let’s analyze the obtained results from fractional calculus point of view. According to (7)

The random walks along the axis of structure (or along stream of plants) were described by the fractional order effective diffusion equation with temporal derivative of fractional order:

18 $$\frac{{\partial }^{1/4}g(x,t)}{\partial {t}^{1/4}}={D}_{xx}^{eff}\frac{{\partial }^{2}g(x,t)}{\partial {x}^{2}}.$$

where $${D}_{xx}^{eff}=\frac{{D}_{1}\,{D}_{3}^{1/4}}{{2}^{3/2}\,{D}_{2}^{1/2}}$$.

Using the expression for *λ*_*y*_ one can obtain by the same way the effective diffusion equation for Y direction:

19 $$\frac{{\partial }^{1/2}g(x,t)}{\partial {t}^{1/2}}={D}_{yy}^{eff}\frac{{\partial }^{2}g(x,t)}{\partial {y}^{2}}.$$

where $${D}_{yy}^{eff}=\frac{{D}_{2}}{2{D}_{3}^{1/2}}$$.

So it is possible to obtain the asymptotic for mean square displacements for different directions (15–17) from effective diffusion equations of fractional order such as (18) and (19). – for more details^11,15^. Consequently the fractional calculus^16^ is a method, which is necessary to use for description of mass transfer processes in plants.

## Conclusion

Thus, based on the study of diffusion processes with using the model of a comb structure, the anomalous character of mass transfer processes in living plants with a branched geometric structure is first established — formulas (8–10). These features of mass transfer in plants with a porous structure are occurred due to the geometry of the structure. Namely, it was found to slow character of the diffusion processes along the stem of plants due to leaving in the side branches, the same reason leads to a slower diffusion along the branches of the first generation. If we observe the diffusion current from stream to branches then we find the acceleration of mass transfer in this structure.

The results can be useful for a more complete understanding of the migration of fertilizers within plant systems and the control of these processes, for example, to increase yield. So slowing speed of the migration of fertilizers along the stem of plants will lead to better absorption of nutrients in the stem of plants, which leads to a thickening of the stem. Similarly, faster migration along the branches of second generation to ensure its growth. The migration along the branches of (N − 1) generation to ensure the growth of leafs.

## References

[1] Mandelbrot, B. *The Fractal Geometry of Nature* (W. H. Freeman and Co., 1982).

[2] Isichenko MB (1992). Percolation, statistical topography, and random media. Rev. Mod. Phys..

[3] Mandelbrot, B. *Fractals and Chaos – The Mandelbrot Set and Beyond* (Springer Science & Business Media, 2013).

[4] Uchaikin VV (2003). Anomalous diffusion and fractional stable distributions. Journal of experimental and theoretical physics.

[5] *Applications of fractional calculus in physics* (ed. Hilfer, R.) (World Science, Singapoure, 2000).

[6] Klafter J, Metzler R (2000). The random walk's guide to anomalous diffusion: A fractional dynamics approach. Physics Reports.

[7] Metzler R, Klafter J (2001). Anomalous stochastic processes in the fractional dynamics framework: Fokker-Planck equation, dispersive transport, and non-exponential relaxation. J. Advances in Chem. Physics.

[8] Shklovskii, B. I. & Efros, A. L. *Electronic Properties Semiconductors*, (Springer-Verlag, Berlin 1984).

[9] Bonch-Bruevich, V. L. *et al*. *Electron Theory of Disordered Semi-Conductors* (in Russian) (Nauka Publishers, Мoscow, 1981).

[10] Weiss G, Havlin S (1986). Some properties of random walks on a comb structure. Physica A.

[11] Arkhincheev VE, Baskin EM (1991). Anomalous diffusion and drift in a comb model of percolation clusters. Journal of experimental and theoretical physics.

[12] Arkhincheev VE (2002). Diffusion on random comb structure: effective medium approximation. Physica A.

[13] Arkhincheev VE (2007). Random walks on the model and its generalizations. Chaos..

[14] Zaburdaev VYu, Chukbar KV (2002). Enhanced superdiffusion and finite velocity of Levy flights. Journal of experimental and theoretical physics.

[15] Arkhincheev, V. E. Fick’s generalized law for anomalous diffusion in the multidimensional comb model. *Letters to Journal of experimental and theoretical physics*, **86**, 580–583 (2007).

[16] Samko, S., Kilbas, A. & Marichev, O. Fractional Integrals and Derivatives: *Theory and Applications*. (Taylor & Francis Books, 1993).

